# Beta-Blocker Therapy After Myocardial Infarction

**DOI:** 10.1016/j.jacadv.2024.101582

**Published:** 2025-01-30

**Authors:** Pilar Cataldo Miranda, Danijela Gasevic, Caroline Trin, Dion Stub, Sophia Zoungas, David M. Kaye, Zhomart Orman, Amminadab L. Eliakundu, Stella Talic

**Affiliations:** a School of Public Health and Preventive Medicine, Monash University, Melbourne, Victoria, Australia; b Centre for Global Health, Usher Institute, The University of Edinburgh, Edinburgh, United Kingdom; c Monash Alfred Baker Centre for Cardiovascular Research, Melbourne, Victoria, Australia

**Keywords:** beta-blockers, CVD, myocardial infarction

## Abstract

Historical data strongly supported the benefits of beta-blocker therapy following a myocardial infarction (MI) for its efficacy in reducing mortality and morbidity. However, in the context of the progressive evolution of treatment strategies for MI patients, the apparent benefit of beta-blocker therapy is becoming less clear. In particular, its effectiveness in patients with preserved left ventricular ejection fraction is currently being challenged. Consequently, contemporary guidelines are now varying in their recommendations regarding the role of beta-blocker therapy in post-MI patients. This review aims to summarize and compare the largest and most influential studies from the prereperfusion era to modern practice regarding different health outcomes while highlighting the need for further research to clarify beta-blocker therapy's place in contemporary post-MI management.

## Highlights

• Beta-blocker therapy has been central to myocardial infarction secondary prevention since the 1980s.
• The effect of beta-blocker therapy in patients with preserved left ventricular ejection function remains inconclusive.
• Key uncertainties include duration of treatment, best beta-blocker type, dosage, and patient selection.
• Evidence suggests a more selective, individualized approach for contemporary myocardial infarction management.

Coronary artery disease (CAD) is the most common cardiovascular disease (CVD) and a leading cause of mortality and disability worldwide. In 2022, ischemic heart disease, which includes CAD, accounted for the highest global cardiovascular mortality, with an age-standardized rate of 108.8 deaths per 100,000 people, underscoring its public health burden.^1^ Characterized by reduced or blocked oxygen flow to the heart, ischemic heart disease is chronic and dynamic in nature.^2^ Acute coronary syndrome (ACS), such as acute myocardial infarction (MI), arises from atherosclerotic plaque rupture or erosion.^2^ CAD and MI incur significant health care costs,^1^^,^^3^ projected to surpass US$1 trillion globally by 2030.^1^

Despite significant advancements in MI treatment, several gaps remain, particularly regarding the optimal use and effectiveness of beta-blocker therapy following MI in patients with normal versus reduced heart function (ie, those with left ventricular dysfunction).^4^^,^^5^ While beta-blocker therapy has been a cornerstone in managing MI due to its ability to reduce myocardial oxygen demand, prevent arrhythmias, and improve survival rates,^6^ its effectiveness for patients with preserved left ventricular ejection fraction (LVEF) or without heart failure remains unclear.^5^^,^^7^^,^^8^ Recent reviews have reported mixed findings regarding the long-term effects of beta-blocker therapy^4^^,^^9^, ^10^, ^11^ and have unveiled the fact that most of the evidence suggesting its significant CV mortality and morbidity benefits was derived from randomized controlled trials (RCTs) conducted in the prereperfusion era (Central Illustration).^12^, ^13^, ^14^, ^15^ This presents a critical gap, as current clinical guidelines are based on outdated evidence and lack data from comprehensive RCTs and real-world evidence (RWE) that accurately reflect today’s diverse patient populations seen in clinical practice.^16^ There is a need for up-to-date, broader and more representative studies that provide insights into the effectiveness of beta-blocker therapy^17^ in the context of modern revascularization procedures and newer pharmacotherapies that have improved post-MI outcomes.^18^^,^^19^

**Central Illustration:**
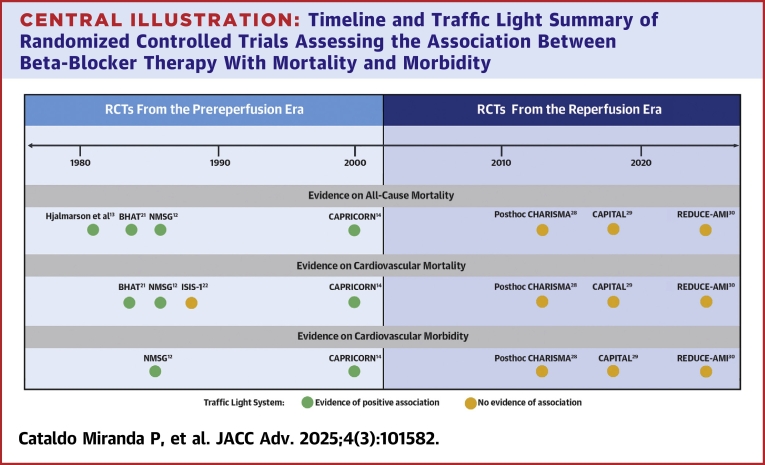
**Timeline and Traffic Light Summary of Randomized Controlled Trials Assessing the Association Between Beta-Blocker Therapy With Mortality and Morbidity** The timeline illustrates included randomized controlled trials during the prereperfusion and reperfusion eras, with a traffic light color coding indicating the type of association between beta-blocker therapy and patient outcomes. The timeline highlights how evidence has evolved over time with the introduction of new treatments and shifts in patient populations, offering insights into the changing clinical context and treatment efficacy. RCTs = randomized controlled trials.

In summary, although beta-blocker therapy has long been a mainstay in post-MI management, several gaps remain in both evidence and practice. Variability in results from studies conducted during the reperfusion era underscores the need for further research. This review aims to summarize the evidence on the effectiveness of beta-blocker therapy across both the prereperfusion and reperfusion eras, highlighting the conflicting results reported in studies from the reperfusion era and appraising possible reasons behind these variations. This state-of-the-art review was informed by a comprehensive search of current literature^20^ for clinical guidelines on post-MI management in the United States, Europe, and Australia, as well as the largest and most influential RCTs from both eras that have played a fundamental role in shaping and revising clinical guidelines. Additionally, the review includes RWE studies from 2014 onward to represent the current clinical setting. Given the controversial and rapidly growing nature of this field, a limited number of observational studies are included in this review.

## Beta-blocker therapy in the pre-reperfusion era

### All-cause mortality

Several RCTs from the 1980s consistently demonstrated that beta-blocker therapy significantly reduces all-cause mortality in post-MI patients, with reductions ranging from 26% to 42.5% (Table 1).^12^^,^^13^^,^^21^ For example, the NMSG (Norwegian Multicentre Study Group), which involved 1,884 patients, reported a 42.5% reduction in all-cause mortality at 33 months in the beta-blocker group compared to the placebo (*P* = 0.03).^12^ Similarly, an RCT comparing intravenous metoprolol to placebo in acute MI patients showed a 36% reduction in all-cause mortality in the beta-blocker group (*P* < 0.03).^13^

**Table 1:** Randomized Controlled Trials Assessing Long-Term Use of Beta-Blocker Therapy and Health Outcomes

First Author or Study Name	Country	Year	Sample Size	Inclusion Criteria	Beta-Blocker	Control	Mean Follow-Up Period	All-Cause Mortality	CV Mortality	CV Morbidity
Hjalmarson et al,^a^^,^^13^	Sweden	1981	1,395	Post-MI, aged 40-74 y	Metoprolol (IV and oral)	Placebo	3 mo	36% reduction	-	-
BHAT^a^^,^^21^	USA	1982	3,837	Post-MI, aged 30-69 y	Propranolol (IV and oral)	Placebo	25 mo	26% reduction	2.3% reduction	-
NMSG^a^^,^^12^	Norway	1982	1,884	Post-MI, aged ≤75 y	Timolol (oral)	Placebo	17 mo	30.6-42.5% reduction	40.4% reduction	28.4-54.4% reduction
ISIS-1^a^^,^^22^	UK	1986	16,027	Post-MI	Atenolol (IV and oral)	Placebo	20 mo	-		-
CAPRICORN^a^^14^	17 countries	2001	1,959	Post-MI, ≥18 y, with LVEF <40%	Carvedilol	Placebo	16 mo	23% reduction	25% reduction	29% reduction
Post-hoc CHARISMA^28^	32 countries	2014	4,772	Post-MI	Use of BB	Nonuser	28 mo			38% reduction recurrent MI
										For stroke and new hospitalizations
CAPITAL^29^	Japan	2018	801	Post-STEMI with PCI and LVEF ≥60%	Carvedilol	Placebo	47 mo			
REDUCE-AMI^30^	Sweden, Estonia, New Zealand	2024	5,020	Post-MI LVEF ≥50% revascularization	Metoprolol and bisoprolol	Nonusers	42 mo			For recurrent MI and new CV hospitalizations

BHAT = β-Blocker Heart Attack Trial; CAPRICORN = Carvedilol Post-Infarct Survival Control in LV Dysfunction; CAPITAL = Carvedilol Post-Intervention Long-Term Administration in Large-scale; CHARISMA = Clopidogrel for High Atherothrombotic Risk and Ischemic Stabilization, Management, and Avoidance; CV = cardiovascular; ISIS-1 = First International Study of Infarct Survival; LVEF = left ventricular ejection fraction; IV = intravenous; MI = myocardial infarction; mo = months; NMSG = Norwegian Multicentre Study Group; PCI = percutaneous coronary intervention; RCT = randomized controlled trial; REDUCE-AMI = Randomised Evaluation of Decreased Usage of Beta-blockers After Acute Myocardial Infarction; STEMI = ST-segment elevation myocardial infarction.

Traffic light summary: Evidence of a positive association ; No evidence of association ; Evidence of a negative association .

a Major RCTs from the prereperfusion era that informed clinical guidelines.

BHAT (β-Blocker Heart Attack Trial), reported a 7.2% mortality rate in the propranolol group, compared to 9.8% in the placebo group, reflecting a significant reduction in mortality (*P* < 0.005).^21^ Additionally, the CAPRICORN (Carvedilol Post-Infarct Survival Control in LV Dysfunction) trial, which included nearly 2,000 patients with LVEF ≤40%, showed a 23% reduction in all-cause mortality (HR: 0.77; 95% CI: 0.60-0.98).^14^ Lastly, Freemantle et al's meta-analysis of 31 RCTs and over 24,000 participants, reported a 23% reduction in long-term mortality risk (OR: 0.77; 95% CI: 0.69-0.85) and concluded that beta-blocker therapy reduced 1.2 events of all-cause mortality.^15^ Moreover, the authors reported that the number needed to treat with beta-blocker therapy to prevent one death was substantially lower than with antiplatelet therapy (42 vs 292), suggesting that beta-blocker therapy was more effective.^15^

### Cardiovascular mortality

Some RCTs from the prereperfusion era reported that beta-blocker therapy was associated with a reduced risk of CV mortality. The NMSG trial reported that beta-blocker therapy reduced sudden cardiac death by 40.4% in all patients (*P* < 0.001), with specific reductions of 31% in patients aged 70 to 75 years, 45.8% in those aged 65 to 69, and 39.8% in those under 65 (all *P* < 0.05).^12^ Similarly, the BHAT trial reported lower CV mortality rates in the propranolol group (6.6% vs 8.9%, *P* < 0.01)^21^ and the CAPRICORN trial showed that fewer patients in the carvedilol group experienced CV death compared to the placebo (HR: 0.75; 95% CI: 0.58-0.96).^14^ In contrast, the ISIS-1 (First International Study of Infarct Survival), a pragmatic RCT involving 16,027 people, found no significant reduction in CV mortality risk among patients who received beta-blocker therapy (*P* = 0.07).^22^

### Cardiovascular morbidity

Evidence on CV morbidity before the reperfusion era was limited, with most studies focusing primarily on reinfarction events. The NMSG trial, for instance, found that timolol therapy reduced the risk of reinfarction by 30.2% in patients younger than 65 years, 28.4% in patients 65 to 69 years of age, and 54.4% in patients 70 to 75 years old (*P* < 0.01).^12^ Similarly, the CAPRICORN trial reported reductions in nonfatal recurrent MI by 41% (HR: 0.59; 95% CI: 0.39-0.90).^14^ Finally, in their meta-analysis, Freemantle et al reported that beta-blocker therapy was associated with a reduction of 0.9 events of recurrence of MI per 100 patients (Table 2).^15^

**Table 2:** Meta-Analyses Assessing Long-Term Use of Beta-Blocker Therapy and Health Outcomes

First Author	Year	Included Studies	Sample Size	Inclusion Criteria	Follow-Up Period	All-Cause Mortality	CV Mortality	CV Morbidity
Freemantle et al,^15^	1999	31 RCT	24,974	RCTs without crossover, examining the effectiveness of BB versus placebo/no treatment in post-MI patients	6-48 mo	Reduction of 1.2 deaths per 100 patients	-	Reduction of 0.9 recurrence MI events per 100
Bangalore et al,^26^	2014	60 RCT	102,003	RCTs comparing BB with placebo/no treatment/other active in post-MI patients with preserved LVEF	Hospital stay-72 mo	-	13% reduction in prereperfusion era	22-28% reduction in recurrent MI
							In reperfusion era	For stroke in prereperfusion era
Huang et al,^45^	2015	10 observational studies	40,873	Studies assessing post-MI and PCI patients	6-48 mo	45% reduction		
Misumida et al,^46^	2016	7 observational studies	10,857	STEMI patients with preserved LVEF, PCI and median follow-up ≥6 mo	6-62 mo	21% reduction	-	-
Dahl Aarvik et al,^47^	2019	16 observational studies	189,385	Post-MI where none or only a minority had LVEF <40% at baseline	6-62 mo	26% reduction	-	-
Safi et al,^11^	2019	63 RCTs	85,550	RCTs assessing BB versus placebo/no intervention in people with suspected or diagnosed MI	6-60 mo	7% reduction	10% reduction	For MACE and recurrent MI
Safi et al,^8^	2021	25 RCTs	22,423	RCTs assessing BB versus placebo/no treatment in patients post-MI without heart failure and LVEF >40%	9-60 mo	19% reduction	27% reduction	28% reduction in MACE 24% reduction in recurrent MI
Maqsood et al,^10^	2021	11 observational studies, 1 RCT	32,108	STEMI patients, PCI	6-56 mo	36% reduction	-	For MACE
Liang et al,^9^	2022	29 observational studies	242,013	Post-MI, median follow-up ≥6 mo, none or only a minority of patients had LVEF <40% at baseline.	6-62 mo	33% reduction	38% reduction	for recurrent MI, new hospitalization, stroke, and MACE in the Asian population
								13.2% reduction of MACE in the Caucasian population
Hu et al,^5^	2022	14 observational studies, 1 RCT	205,672	ACS without heart failure or left ventricular systolic dysfunction.	12-60 mo	34% reduction		for MACE, recurrent MI, or stroke
Kim et al,^4^	2022	5 observational studies	217,532	Post-MI, no heart failure at baseline.	12-42 mo		-	-

ACS = acute coronary syndrome; MACE = major adverse cardiac events; other abbreviations as in Table 1.

Traffic light summary: Evidence of a positive association ; No evidence of association ; Evidence of a negative association .

For decades, clinical guidelines have extrapolated these results to make recommendations in an ever-changing clinical setting. Yet these recommendations have been constrained by several factors. First, most RCTs were conducted in controlled environments with strict inclusion and exclusion criteria, which may not capture the full spectrum of patients encountered in routine care.^23^ Second, the populations studied often included patients with large MI^14^^,^^22^ and left ventricular systolic dysfunction,^14^ which may not reflect the current clinical setting. Additionally, various study limitations, such as high risk of bias,^12^^,^^13^^,^^21^^,^^22^ operational definitions of beta-blocker therapy mainly as intravenous treatment,^13^^,^^21^^,^^22^ lack of subanalyses by MI type or LVEF,^12^, ^13^, ^14^^,^^21^^,^^22^ and variation in beta-blocker agents and dosages,^12^, ^13^, ^14^^,^^21^^,^^22^ have further complicated the interpretation of results.

**Figure 1:**
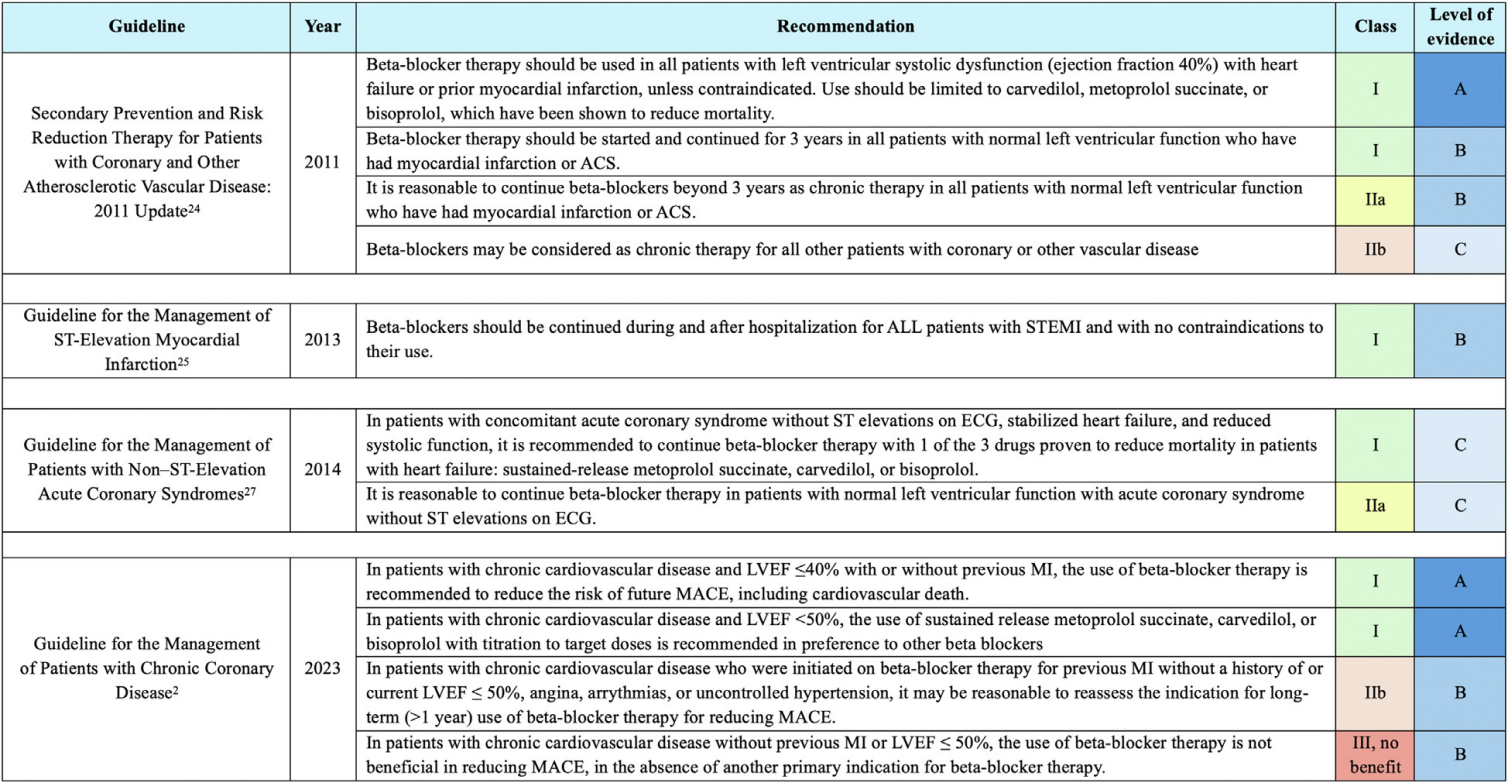
**American Heart Association/American College of Cardiology Guidelines: Long-Term Beta-Blocker Therapy for Post-Myocardial Infarction Prevention** This figure summarizes key updates in the AHA/ACC guidelines for beta-blocker use in secondary prevention after MI, from 2011 to 2023. It shows evolving recommendations, class of recommendation, and level of evidence, including various patient populations such as those with left ventricular dysfunction, heart failure, and CAD. The figure also highlights changes in treatment duration, patient selection, and specific beta-blocker medications. ACS = acute coronary syndrome; AHA/ACC = American Heart Association/American College of Cardiology; CAD = coronary artery disease; LVEF = left ventricular ejection fraction; MACE = major adverse cardiac events; MI = myocardial infarction; STEMI = ST-segment elevation myocardial infarction.

Despite these limitations, prereperfusion evidence on beta-blocker therapy strongly influenced the American Heart Association/American College of Cardiology Foundation (AHA/ACCF) clinical guidelines until 2013 (Figure 1). For example, the 2011 Secondary Prevention and Risk Reduction Therapy for Patients With Coronary and Other Atherosclerotic Vascular Diseases guideline recommended beta-blocker therapy for all patients with LVEF <40% and post-MI patients regardless of LVEF, even beyond 3 years.^24^ Similarly, the 2013 AHA/ACCF Management of ST-Elevation Myocardial Infarction (STEMI) guidelines suggested beta-blocker therapy for all hospital discharged patients without contraindications.^25^ However, a 2014 meta-analysis by Bangalore et al, which included over 60 RCTs and 102,003 patients, revealed a significant interaction between beta-blocker therapy and reperfusion status (Table 2).^26^ Beta-blocker therapy reduced the risk of all-cause mortality (incident rate ratio [IRR]: 0.86; 95% CI: 0.79-0.94) and recurrence of MI (IRR: 0.78; 95% CI: 0.62-0.97) in the prereperfusion era.^26^ Yet, in the reperfusion era, where over 50% of patients received thrombolytics or underwent revascularization, beta-blocker therapy did not significantly reduce mortality (IRR: 0.98; 95% CI: 0.92-1.05).^26^ As a result, the 2014 AHA/ACCF Management of Patients with Non–ST-Elevation (NSTEMI) Acute Coronary Syndromes guidelines strongly recommended beta-blocker therapy in patients with reduced LVEF^27^ and suggested continuing therapy beyond 3 years for patients with normal LVEF and no angina, given the unclear evidence for long-term benefits in this group.^27^ The Bangalore et al^26^ findings highlighted that up until 2014, most evidence regarding the effectiveness of beta-blocker therapy pertained to patients with reduced LVEF, prompting questions regarding the effectiveness of beta-blocker therapy in more stable patients with preserved LVEF and the optimal duration of its use.

### Beta-blocker therapy in the reperfusion era

The observed clinical benefits of beta-blocker therapy during the prereperfusion era are attributed to complex mechanisms of the beta-adrenoceptor blockade, including a reduction in myocardial work and thus myocardial oxygen consumption, reduced wall stress leading to less remodeling and anti-arrhythmic actions.^2^^,^^7^^,^^11^ However, its benefits appear less significant within the framework of modern practice. This is mainly due to the introduction of early reperfusion therapies with newer thrombolytic agents and invasive strategies, such as the percutaneous coronary intervention (PCI) in the late 1970s.^7^ The emergence of this reperfusion therapy increased rates of myocardial salvage and decreased ischemic times, leading to reduced mortality rates for MI patients.^19^ Furthermore, new progressive pharmacotherapies, such as angiotensin-converting enzyme inhibitors facilitated the improvement of LVEF by mitigating ventricular remodeling, while statins enhanced survival by stabilizing atherosclerotic plaques and reducing the risk of recurrent CV events post-MI.^18^ In addition, these therapies have also enhanced patients’ quality of life while avoiding the multiple side effects commonly associated with beta-blocker therapy.^7^

### All-cause mortality

Contemporary evidence from RCTs indicates no association between using beta-blocker therapy and reduced all-cause mortality (Table 1). For example, a post hoc analysis from the CHARISMA (Clopidogrel for High Atherothrombotic Risk and Ischemic Stabilization, Management, and Avoidance) trial found that beta-blocker therapy in patients with previous MI, but no heart failure, did not affect mortality rates (HR: 0.77; 95% CI: 0.52-1.14).^28^ Similarly, the CAPITAL-RCT (Carvedilol Post-Intervention Long-Term Administration in Large-scale Randomized Controlled Trial) with a median follow-up of 3.9 years, reported no significant difference in the 3-year incidences of composite outcomes including all-cause mortality, MI, hospitalization for heart failure, and hospitalization for ACS between the carvedilol group and no beta-blocker therapy group (HR: 0.75; 95% CI: 0.47-1.16).^29^ Moreover, there was no significant difference in LVEF at 1 year between the 2 groups.^29^

The findings from the recently published REDUCE-AMI (Randomised Evaluation of Decreased Usage of Beta-blockers After Acute Myocardial Infarction) trial, a pragmatic RCT involving patients from Sweden, Estonia, and New Zealand who underwent revascularization and had an LVEF ≥50% after acute MI, found no significant difference in all-cause mortality between those who received beta-blocker therapy and those who did not (HR: 0.96; 95% CI: 0.79-1.16).^30^ This trial is the first to provide insights specifically on patients with preserved LVEF (≥50%). However, the ABYSS (Assessment of β-blocker interruption 1 Year after an uncomplicated myocardial infarction on Safety and Symptomatic cardiac events requiring hospitalization) trial, a noninferiority study conducted in France, found that interrupting beta-blocker therapy 1 year after an uncomplicated MI did not reduce the risk of a composite outcome (death, recurrent MI, nonfatal stroke, or hospitalization for cardiovascular events) over a median follow-up of 3 years (HR: 1.16; 95% CI: 1.01-1.33; *P* = 0.44 for noninferiority).^31^ In addition, the trial reported that the interruption of beta-blocker therapy did not result in an improvement in quality of life.^31^

Worldwide, national population-level data sets and clinical registries provide extensive but inconsistent observational data (Table 3). In South Korea, a study utilizing claims data from the Korean National Health Insurance Service (KNHIS) involving 81,752 patients reported that 66% were prescribed beta-blockers at discharge.^32^ Over a mean 2-year follow-up, regular use of beta-blocker therapy reduced the risk of composite events, including death, MI, and stroke by 36% compared to nonuse (HR: 0.64; 95% CI: 0.56-0.73).^32^ Another Korean study of 28,970 patients postrevascularization after acute MI, reported that over a mean follow-up period of 3.5 years patients receiving beta-blocker therapy for over 1 year had a significantly lower risk of all-cause mortality (HR: 0.81; 95% CI: 0.72-0.91) and of composite outcomes (all-cause mortality, recurrent MI, and hospitalization) compared to those receiving beta-blocker therapy for less than 1 year (HR: 0.82; 95% CI: 0.75-0.89).^33^ The benefit persisted beyond 2 years (HR: 0.86; 95% CI: 0.75-0.99) but not beyond 3 years (HR: 0.87; 95% CI: 0.73-1.03).^33^

**Table 3:** Observational Studies Assessing Long-Term Use of Beta-Blocker Therapy and Health Outcomes

Registry	Country	Year	Study Type	Sample Size	Inclusion Criteria	Beta-Blocker Users (%)	Nonusers (%)	Mean Follow-Up Period	All-Cause Mortality	CV Mortality	CV Morbidity
COREA-AMI^34^	Korea	2014	Retrospective	3,019	LVEF ≥50% post-MI and PCI	80.3%	19.7%	3 y	36.7% reduction	53% reduction	For recurrent MI
CORONOR^48^	France	2014	Retrospective	4,184	Post-MI and/or revascularization	79%	21%	2 y	-	36% reduction	-
FAST-MI^37^	France	2016	Prospective	2,679	LVEF >40% post-MI	80.4%	19.6%	5 y			-
MINAP^40^	UK	2017	Retrospective	179,810	LVEF >30% post first-MI, no heart failure	78.5%	21.5%	1 y		-	-
CMS-CCW^44^	USA	2017	Retrospective	90,869	≥65 y/o post-MI	76.2%	23.8%	0.9 y	44% increased risk in diabetic patients	-	-
French Healthcare Databases^38^	France	2018	Retrospective	73,450	25-79 y/o post-MI and revascularization	94.1%	5.9%	3.8 y		-	15% reduction in new hospitalization
CLARIFY^41^	45 countries	2019	Retrospective	32,378	Post-MI and revascularization	77.9%	22.1%	5 y			
KNHIS^32^	Korea	2020	Retrospective	81,752	Post first-MI and PCI	66%	13%	2 y	36% reduction	-	29.5% reduction in recurrent MI
											For stroke
KNHIS^33^	Korea	2020	Retrospective	28,970	Post first MI and revascularization	78.4%	21.6%	3.5 y	19% reduction	-	18% reduction in recurrent MI and hospitalization
KAMIR-NIH^35^	Korea	2021	Retrospective	4,008	1-y ECG follow-up post-MI	79.3%	20.7%	1 y	For LVEF ≥50%	For LVEF ≥50%	-
									51% reduction for LVEF <50%	56% reduction for LVEF <50%	
KAMIR-NIH^49^	Korea	2021	Retrospective	12,200	Post-MI	84%	16%	1 y	19% reduction	16% reduction for LVEF <40%	37% reduction in MACE for LVEF <40% and 31% in 40 < LVEF <50%
										For LVEF ≥50%	For stroke and new hospitalizations. For MACE for LVEF >50%
MIG^36^	Australia	2021	Retrospective	17,562	≥18 y/o with ACS and PCI	83.3%	16.7%	5.3 y	For LVEF >50%	-	-
SWEDEHEART^42^	Sweden	2021	Retrospective	40,697	Post first MI	90.6%	9.4%	4 y	For LVEF ≥50%	-	For LVEF ≥50%
DNPR^43^	Denmark	2021	Retrospective	30,177	30-85 y/o post first MI and revascularization	82%	18%	2.8 y			
ePARIS^39^	France	2023	Retrospective	1,887	STEMI patients free of CV events for 6-mo and LVEF ≥40%	80%	20%	2.7 y	61% reduction	-	-

ECG = electrocardiogram; other abbreviations as in Table 1.

Traffic light summary: Evidence of a positive association ; No evidence of association ; Evidence of a negative association .

Included observational studies used population-level data sets or clinical registry data.

Evidence from the Convergent Registry of Catholic and Chonnam University for Acute MI (COREA-AMI) showed a 36.7% reduction in 3-year all-cause mortality risk for post-MI patients who underwent PCI, had preserved systolic function (LVEF ≥50%), and were treated with beta-blocker therapy (HR: 0.63; 95% CI: 0.46-0.86).^34^ In contrast, analysis of the Korean Acute Myocardial Infarction Registry-National Institutes of Health (KAMIR-NIH) registry reported that the use of beta-blocker therapy at 1-year follow-up after an electrocardiogram (ECG) following an acute MI reduced the 2-year all-cause mortality risk by 51% in patients with a 1-year LVEF <50% (HR: 0.49; 95% CI: 0.26-0.89)^35^ but not in patients with LVEF ≥50% (HR: 1.35; 95% CI: 0.74-2.46).^35^ Similarly, evidence from the Melbourne Interventional Group registry (MIG) involving 17,562 patients with a mean follow-up period of 5.3 years, demonstrated that beta-blocker therapy after PCI significantly reduced the risk of all-cause mortality by 37% in patients with LVEF <35% (HR: 0.63; 95% CI: 0.44-0.91) and by 20% in those with LVEF between 35% and 50% (HR: 0.80; 95% CI: 0.68-0.95) but had no effect in those with LVEF >50% (HR: 1.03; 95% CI: 0.87-1.21).^36^

Results from the Nationwide French registry of Acute ST- and non-ST-elevation Myocardial Infarction (FAST-MI) indicated that beta-blocker therapy within 48 hours of acute MI significantly reduced 30-day mortality (HR: 0.46; 95% CI: 0.26-0.82) but subgroup analyses according to age, sex, type of MI, and LVEF (>50% and 40% to 50%) showed no significant effect on 1-year survival (HR: 0.77; 95% CI: 0.46-1.30) and 5-year survival (HR: 1.19; 95% CI: 0.65-2.18).^37^ Similarly, in a cohort of 73,450 revascularized patients from the French Healthcare Databases, discontinuation of beta-blocker therapy (ie, 4 consecutive months without exposure) was not associated with a higher risk of all-cause mortality (HR: 1.13; 95% CI: 0.94-1.36).^38^ Results of ePARIS registry involving 1,887 STEMI patients with LVEF ≥40% reported a 61% higher risk of mortality in patients without beta-blocker therapy prescription at discharge (HR: 0.39; 95% CI: 0.28-0.55).^39^

However, some studies found no association between beta-blocker therapy and reduced all-cause mortality risk in post-MI patients with preserved LVEF, including the MINAP (Myocardial Ischaemia National Audit Project) (average treatment effect coefficient: 0.07; 95% CI: -0.60-0.75),^40^ the CLARIFY (Prospective Observational Longitudinal Registry of Patients with Stable Coronary Artery Disease) registry (HR: 0.94; 95% CI: 0.84-1.06),^41^ the SWEDEHEART (Swedish Web-system for Enhancement and Development of Evidence-based Care in Heart Disease Evaluated According to Recommended Therapies (HR: 0.89; 95% CI: 0.78-1.01),^42^ and the Danish National Patient Register (absolute risk differences −0.4%; 95% CI: −1.0% to 0.2%).^43^ Additionally, Korhonen et al’s study using the U.S. Center for Medicare & Medicaid Services Medicare Chronic Condition Data Warehouse (CMS-CCW) found that patients adhering only to beta-blocker therapy had a 32% higher mortality risk than those adhering to all recommended secondary prevention medications (angiotensin-converting enzyme inhibitors/angiotensin receptor blockers, beta-blockers, and statins) (HR: 1.32; 95% CI: 1.21-1.44), with a more pronounced effect in diabetic patients (HR: 1.44; 95% CI: 1.28-1.62), particularly in men and younger individuals.^44^

Meta-analyses pooling data from RCTs and observational studies have reported a positive relationship between the use of beta-blocker therapy and risk reduction in all-cause mortality in post-MI patients who underwent revascularization^5^^,^^45^^,^^46^ and had, mostly, preserved LVEF (Table 2).^5^^,^^8^^,^^10^^,^^46^^,^^47^ Yet, a recent meta-analysis of observational cohort studies that included 217,532 patients reported that the use of beta-blocker therapy for 1 year or more did not significantly reduce the risk of premature mortality of MI patients without heart failure compared to those who did not receive beta-blocker therapy (OR: 0.8; 95% CI: 0.56-1.14).^4^

### Cardiovascular mortality

RCT results show no association between beta-blocker therapy and CV mortality risk (Table 1). For instance, post hoc analyses of the CHARISMA trial found no link between beta-blocker therapy and reduced CV mortality risk (HR: 0.85; 95% CI: 0.52-1.40).^28^ Similarly, results from the REDUCE-AMI trial showed no differences in death from CV causes among revascularized patients with LVEF ≥50% (HR: 1.15; 95% CI: 0.72-1.84).^30^

Evidence from observational data is conflicting (Table 3). The French CORONOR registry reported a 36% risk reduction in CV mortality at 2-year follow-up for post-MI patients, with or without revascularization, using beta-blocker therapy (HR: 0.64; 95% CI: 0.42-0.98).^48^ Similarly, results from the COREA-AMI registry showed a significant risk reduction at 3 years of follow-up for post-MI patients with LVEF ≥50% who underwent PCI (HR: 0.47; 95% CI: 0.32-0.70).^34^ Analyses using the KAMIR-NIH registry reported reduced risk in patients with LVEF <50% (HR: 0.44; 95% CI: 0.21-0.94^35^ and HR: 0.74; 95% CI: 0.57-0.96)^49^ but not in those with LVEF ≥50% at 1-year follow-up (HR: 1.21; 95% CI: 0.56 to 2.62^35^ and HR: 1.16; 95% CI: 0.91-1.48).^49^ Most observational studies reported no association between beta-blocker therapy and reduced CV mortality risk in revascularized patients,^41^ without heart failure,^40^ preserved LVEF,^35^^,^^37^ and optimally treated with other secondary CV prevention drugs.^43^

Meta-analyses present conflicting results (Table 2). Safi et al’s meta-analysis of 63 RCTs involving 85,550 participants reported a 10% risk reduction in CV death over a mean follow-up period of 12.9 months (relative risk [RR]: 0.90; 98% CI: 0.83-0.98).^11^ A 2021 meta-analysis based on 25 RCTs involving 22,423 participants reported a 27% risk reduction in CV mortality over 28.8 months (RR: 0.73; 98% CI: 0.61-0.88).^8^ Similarly, Liang et al showed a 38% CV death risk reduction when using beta-blocker therapy (HR: 0.62; 95% CI: 0.49-0.78), with significant results for both Caucasian and Asian populations.^9^ However, meta-analyses by Huang et al and Hu et al focusing on patients who underwent PCI^45^ and without heart failure and preserved LVEF,^5^ reported no association between beta-blocker therapy and CV mortality.^5^^,^^45^

### Cardiovascular morbidity

RCT results on beta-blocker therapy for CV morbidity are conflicting (Table 1). A post hoc analysis of the CHARISMA trial found a 38% reduction in recurrent MI (HR: 0.62; 95% CI: 0.39-1.00), but no effect on stroke (HR: 2.13; 95% CI: 0.92-4.92) or new hospitalizations (HR: 1.40; 95% CI: 0.84-2.34).^28^ Similarly, the results from the recently published REDUCE-AMI trial showed no association between beta-blocker therapy and recurrent MI (HR: 0.96; 95% CI: 0.74-1.24) or new CV hospitalizations for heart failure (HR: 0.91; 95% CI: 0.50-1.66).^30^ On the contrary, the ABYSS trial reported that patients who interrupted beta-blocker therapy had a numerical increase in the risk of recurrent angina and other CV conditions that led to hospitalizations and coronary procedures.^31^ However, no hypothesis testing was performed for these secondary outcomes in this trial which means that the observed differences between groups cannot be concluded as statistically significant. Additionally, because the ABYSS trial aimed to demonstrate noninferiority rather than superiority, the conclusions are focused on whether the interruption of beta-blocker therapy met noninferiority criteria, not whether it led to worse clinical outcomes.^31^ This distinction in trial design is key to understanding why these results cannot be easily compared to other RCTs that may have tested superiority.

Observational studies also show mixed results (Table 3). Evidence from the KAMIR-NIH registry showed that beta-blocker therapy was associated with a significant risk reduction for major adverse cardiac events (MACE) in patients with LVEF <50%, particularly in those with LVEF <40% (HR: 0.84; 95% CI: 0.72-0.97).^49^ However, no association was observed for patients with LVEF ≥50% (HR: 1.16; 95% CI: 0.91-1.48).^49^ Results from the Korean National Health Insurance Service registry reported that patients receiving beta-blocker therapy for at least 1 year had an 18% lower risk of combined all-cause death, recurrent MI, or hospitalization for new heart failure (HR: 0.82; 95% CI: 0.75-0.89)^33^ but had no significant difference in the risk of recurrent MI alone (HR: 0.82; 95% CI: 0.67-1.01) or hospitalization for new heart failure alone (HR: 0.92; 95% CI: 0.73-1.17).^33^ Won et al, using the same registry data, found that regular use of beta-blocker therapy was associated with a 29.5% reduced risk of MI (HR: 0.71; 95% CI: 0.06-0.84)^32^ but it did not affect the risk of stroke (HR: 1.05; 95% CI: 0.85-1.30).^32^ Additionally, the French Healthcare Database indicated that discontinuing beta-blocker therapy after 1 year was associated with a 17% increased risk of CVD-related hospital admissions (among post-MI patients under 80 years of age who underwent revascularization (HR: 1.17; 95% CI: 1.01-1.35).^38^

Despite these favorable results, other studies have reported no association between beta-blocker therapy and reduced risk of the recurrence of MI and stroke in post-MI patients with LVEF ≥50%,^34^^,^^42^ who underwent revascularization^34^^,^^41^ and were optimally treated with other secondary cardiovascular prevention medications.^43^

Results from meta-analyses vary (Table 2). Maqsood et al reported no association between beta-blocker therapy and risk reduction of MACE (OR: 0.87; 95% CI: 0.70-1.08),^10^ while Safi et al reported no effect on MACE and stroke (RR: 0.81; 97.5% CI: 0.43-1.52).^11^ However, in their 2021 meta-analysis, Safi et al reported that beta-blocker therapy reduced the risk of MACE by 28% (RR: 0.72; 97.5% CI: 0.62-0.83) and the recurrence of MI by 24% (RR: 0.76; 98% CI: 0.67-0.86) in patients with LVEF >40%.^8^ Liang et al reported significant risk reduction for MACE in the Caucasian population (HR: 0.88; 95% CI: 0.79-0.97) but not in the Asian population (HR: 0.89; 95% CI: 0.69-1.15).^9^ In addition, the authors reported no association between beta-blocker therapy and the recurrence of MI (HR: 0.93; 95% CI: 0.78-1.11) and stroke (HR: 0.94; 95% CI: 0.79-1.12).^9^ Similarly, Hu et al reported no association between beta-blocker therapy and MACE (OR: 0.98; 95% CI: 0.80-1.21), recurrent MI (OR: 1.10; 95% CI: 0.80-1.50), and stroke (OR: 0.98; 95% CI: 0.37-2.59) in patients with preserved LVEF function.^5^

### Why conflicting results?

The variability in the effectiveness of beta-blocker therapy results from differences in study design, methodologies, and statistical approaches, leading to inconsistent outcomes and selection biases. Specific reasons are discussed below.

### Limitations of contemporary RCTs

Even though RCTs are designed to be unbiased and are regarded as the highest level of evidence,^50^ these types of studies still have limitations. The CAPITAL, REDUCE-AMI, and ABYSS trials have several limitations which raise important considerations for future research on beta-blocker therapy following an MI and broader cardiovascular contexts. Watanabe’s study, being underpowered, lacked sufficient statistical power to detect meaningful differences, highlighting the need for adequately powered trials to assess beta-blocker withdrawal or continuation in various patient populations.^29^ The open-label design used in these trials may have introduced potential bias, as both patients and physicians knew the treatment allocation.^29^, ^30^, ^31^ This may have led to treatment crossover, especially in the no-beta-blocker or interruption groups, diluting the effects of the intervention. To mitigate this, future studies should aim for double-blind designs, or at the very least, strict protocols to minimize crossover and include both per-protocol and intention-to-treat analyses to better capture the impact of treatment adherence and protocol deviations.

Furthermore, neither the REDUCE-AMI nor CAPITAL trial adequately addressed the effects of beta-blocker therapy in patients with mid-range LVEF (40% to 50%).^29^^,^^30^ While the ABYSS trial included patients with LVEF ≥40%, its findings may not be generalizable beyond Europe due to regional differences in health care systems and clinical practices.^31^ To address this limitation, future research should consider multicenter, multicounty RCTs to provide more globally relevant results and help inform worldwide clinical guidelines on beta-blocker use after MI.

Another limitation is the relatively short follow-up duration in many RCTs, which may fail to capture long-term outcomes like recurrent angina, heart failure progression, or other cardiovascular events. Extending follow-up periods in future studies will provide better insights into the long-term effects of beta-blocker therapy, including its impact on mortality, recurrent events, and quality of life, particularly in diverse patient subgroups.

### Limitations of RWE studies

#### Variability in research design and methodologies

RWE studies often vary in inclusion and exclusion criteria, as well as in their operational definitions of exposure and outcome. These differences can impact baseline characteristics of study populations, leading to heterogeneity in results. For example, some studies did not distinguish between STEMI and NSTEMI patients,^32^^,^^33^^,^^38^ while others included patients with and without reperfusion therapy.^37^ Additionally, unlike RCTs, RWE encounters significant challenges in mitigating biases that may affect results, as in observational studies associations rather than causal relationships are typically established.^33^ Despite covariate adjustments to control for measured confounding variables, unmeasured confounding variables, such as contraindications to beta-blocker therapy^32^, ^33^, ^34^, ^35^^,^^44^ (ie, bronchial asthma, arteriosclerosis obliterans, and behavioral and anthropometric factors) may still influence results.^33^^,^^34^ In addition, some studies lacked information regarding the type of MI, angiographic severity,^32^^,^^33^^,^^38^ or the type of beta-blocker being used,^33^^,^^34^^,^^36^^,^^38^^,^^40^ all of which may lead to high heterogeneity due to different pathophysiology and pharmacological properties among drug classes. Another source of bias derives from not knowing the reasons for beta-blocker use, which typically vary between patients. For instance, individuals receiving long-term beta-blocker therapy might have less severe risk profiles than those ceasing beta-blocker therapy during the follow-up period.^32^^,^^33^^,^^36^ Lastly, some studies had small sample sizes, which increases the risk of type B error, potentially underestimating the benefit of beta-blocker therapy.^37^

#### Variability in statistical approaches

Different statistical analyses were used across studies. Some did not adjust for LVEF,^32^^,^^33^^,^^38^ while others conducted matched population analyses^32^^,^^37^^,^^48^^,^^49^ which cannot be generalized. Additionally, several studies measured composite outcomes,^32^^,^^33^^,^^38^^,^^41^, ^42^, ^43^^,^^49^ which pose challenges in interpretation due to variations in severity or benefit among each of the outcome components.

#### Intrinsic limitations of registry data

As most data were derived from observational registries, event detection and patient follow-up were less rigorous compared to RCTs, resulting in a higher risk of underreporting specific outcomes.^32^^,^^34^^,^^39^^,^^40^^,^^49^ Even more, not all registries captured every relevant covariate, increasing the risk of unmeasured variables and residual confounding.^32^^,^^33^^,^^35^, ^36^, ^37^^,^^40^, ^41^, ^42^^,^^44^^,^^48^^,^^49^ Moreover, not all specified whether the medication was being used before the MI event,^34^^,^^40^^,^^43^ which may influence adherence and consequently, the outcome. Additionally, in some studies, treatment was determined based on prescriptions filled, which may not reflect actual medication use.^33^, ^34^, ^35^, ^36^^,^^38^^,^^40^^,^^42^^,^^44^^,^^48^^,^^49^ Thus, the resulting estimates may be incorrect due to this assumption. Adherence to medication regimes may significantly impact the effectiveness of beta-blocker therapy and contribute to varied results.^29^ RCTs generally report higher adherence due to strict protocols, continuous monitoring, and controlled environments that are not typically found in real-world settings. Factors such as selective inclusion criteria, enhanced support systems, financial incentives, and simplified treatment regimens in RCTs help create an environment where adherence is often better than in RWE studies.^16^ Finally, it is presumed that the prescribed dose is ideal,^34^^,^^49^ though this may not always be the case.

### Limitations of meta-analyses

Meta-analyses were limited not only by the characteristics of the included observational studies mentioned earlier but also by their respective design and methodology. For instance, Dahl Aarvik et al’s study was limited by residual confounding, small study effect, and publication bias.^47^ Residual confounding refers to the influence of unmeasured or inadequately adjusted variables which could skew the observed associations, while small study effects refer to the tendency for smaller studies to report exaggerated effect sizes, combined with publication bias (ie, the selective publication of positive results). These issues highlight the need for future meta-analyses to employ robust statistical methods, such as sensitivity analyses and funnel plots, to assess biases.

Bangalore et al's meta-analysis did not differentiate between studies from the prereperfusion and reperfusion eras, leading to a higher risk of bias.^26^ The meta-analysis included a substantial proportion of prereperfusion era studies with a high risk of bias and may not reflect current clinical practice, weakening its relevance. Similarly, meta-analyses conducted by Kim et al,^4^ Hu et al,^5^ Liang et al,^9^ and Huang et al^45^ had a high degree of heterogeneity and their findings were largely impacted by confounding factors, such as comorbidities, concomitant treatments, and baseline population characteristics. This variability calls for subgroup analyses or meta-regressions to better understand and control for these factors.

The Hu et al^5^ methodology may have been underpowered for certain outcomes, while the Safi et al^5^^,^^11^ studies recognized a high risk of bias in included RCTs which may have led to overestimated benefits and underestimated harms. Moreover, Safi et al^11^ did not clearly define “long-term” beta-blocker use, arbitrarily classifying groups as using medication for less than or more than 3 months, which introduces further ambiguity and limits the ability to make reliable conclusions.

Overall, the clinical heterogeneity among the studies included in these meta-analyses, stemming from differences in design, population, interventions, and outcomes, presents a significant barrier to making valid comparisons or drawing firm conclusions. Future meta-analyses should incorporate standardized definitions of key variables, clearer definitions of time periods, use advanced statistical techniques to assess and manage heterogeneity, and ensure better comparability among included studies.

### Implications on guidelines

**Figure 2:**
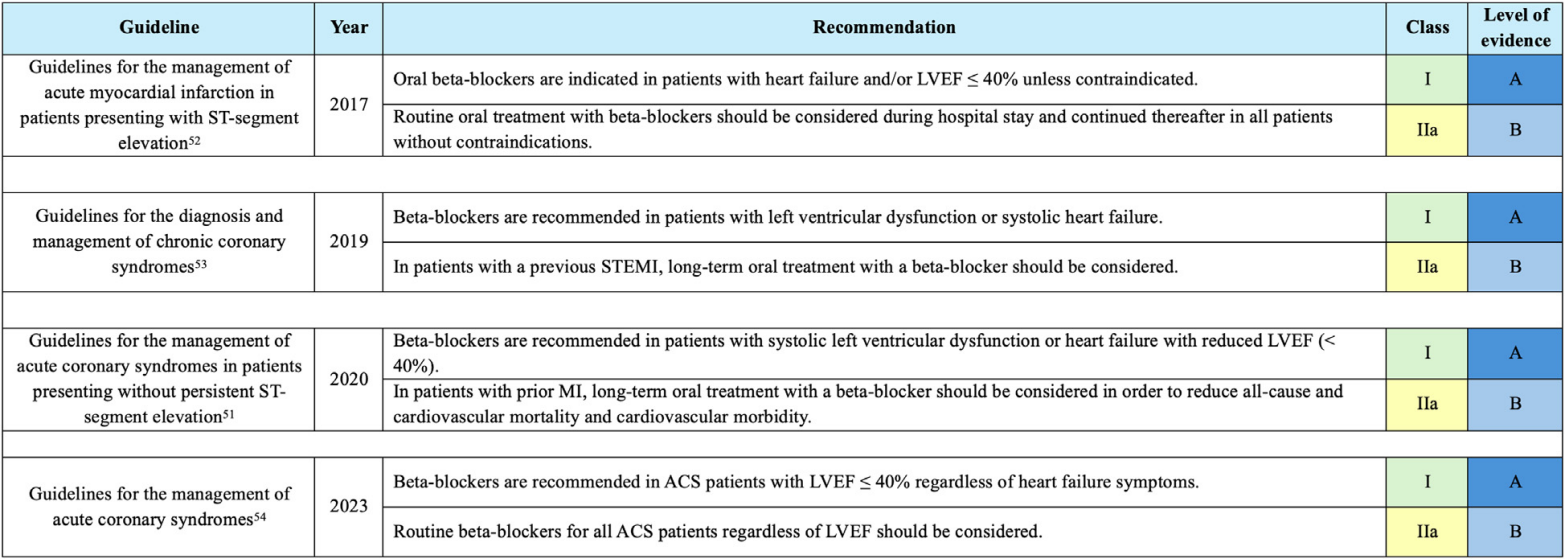
**ESC Guidelines: Long-Term Beta-Blocker Therapy for Post-Myocardial Infarction Prevention** This figure illustrates key updates in the ESC guidelines on beta-blocker therapy for secondary prevention following MI, from 2017 to 2023. It highlights evolving recommendations for patients with LVEF ≤40%, heart failure, and a history of MI, as well as the class of recommendation and level of evidence for beta-blocker use in ACS and chronic coronary syndromes. The figure also reflects a shift toward broader use of beta-blockers in ACS patients, regardless of heart failure status. ESC = European Society of Cardiology; other abbreviations as in Figure 1.

**Figure 3:**
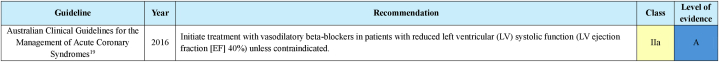
**NHFA/CSANZ Guidelines: Long-Term Beta-Blocker Therapy for Post-Myocardial Infarction Prevention** This figure summarizes the 2016 NHFA and CSANZ guidelines on beta-blocker use for secondary prevention after MI. It emphasizes initiating beta-blockers in patients with reduced LVEF (≤40%) unless contraindicated, with a class IIa recommendation and level a evidence for this high-risk group. NHFA/CSANZ = National Heart Foundation of Australia & Cardiac Society of Australia and New Zealand; other abbreviations as in Figure 1.

Current evidence on beta-blocker therapy, largely derived from observational studies,^32^, ^33^, ^34^, ^35^, ^36^, ^37^, ^38^, ^39^, ^40^, ^41^, ^42^, ^43^, ^44^^,^^48^^,^^49^ is conflicting,^32^^,^^34^, ^35^, ^36^, ^37^, ^38^^,^^40^, ^41^, ^42^, ^43^, ^44^^,^^49^ leading to variability in clinical guidelines. The European Society of Cardiology (ESC) recommends beta-blockers for patients with STEMI or NSTEMI and reduced LVEF (<40%)^51^, ^52^, ^53^ and suggests long-term treatment for those with a history of prior MI, though without specifying duration.^52^^,^^53^ Its current guideline for ACS management recommends beta-blocker use in patients with LVEF ≤40% but does not distinguish between STEMI and NSTEMI or provide guidance on long-term therapy (Figure 2).^54^ Similarly, the current AHA/ACC guideline for chronic coronary disease management recommends beta-blocker therapy for patients with LVEF <50% but advises reassessing the indication of treatment at 1-year post-MI for patients without reduced LVEF. They do not recommend long-term therapy for those with LVEF >50%.^2^ The National Heart Foundation of Australia & Cardiac Society of Australia and New Zealand (NHFA/CSANZ) Guidelines for ACS management (Figure 3) recommends beta-blocker therapy for patients with LVEF <40%, without differentiating between STEMI and NSTEMI, and lacks clear guidance for those with LVEF >40%.^19^

These variations in guidelines contribute to differing prescription rates globally. For instance, 93% of eligible patients in Europe receive beta-blockers, compared to just 56.5% in South-East Asia.^55^ In Italy, more than one-half of MI patients were prescribed beta-blocker therapy, though prescription rates have declined over time.^56^ In Australia, according to the Australian Bureau of Statistics, 33% of adults with CVD used beta-blockers in 2017 to 2018,^57^ though adherence rates are suboptimal, with only 73% continuing therapy after 12 months.^58^

Several ongoing trials are expected to provide greater clarity on the optimal use of beta-blocker therapy in the context of modern reperfusion therapies. The following RCTs are assessing the effectiveness of beta-blocker therapy in post-MI patients with preserved LVEF (≥40%): the DANBLOCK (Danish trial of beta-blocker treatment after myocardial infarction without reduced ejection fraction),^59^ the BETAMI (BEtablocker Treatment After acute Myocardial Infarction in revascularized patients without reduced left ventricular ejection fraction),^60^ and the REBOOT (tREatment with Beta-blockers after myOcardial infarction withOut reduced ejection fracTion) trial.^61^ The results from these studies are anticipated to inform and potentially improve current clinical guidelines.

## Conclusions

This review has demonstrated that although beta-blocker therapy has been a fundamental part of secondary prevention post-MI for decades, recent evidence suggests its effectiveness in patients with preserved LVEF is inconclusive. Variability across observational studies, RCTs, and meta-analyses, due to differences in study populations, limits comparability. An umbrella review focused on populations with similar LVEF could improve generalizability and inform future research and clinical guidelines.

Many questions remain, including the most useful type of beta-blocker agent, optimal dosage, which patients benefit most, and its role across different ACS types. The role of beta-blocker therapy in contemporary MI management is evolving, with evidence suggesting their use should be more selective and tailored to high-risk patients. Ongoing trials are expected to clarify their optimal use in the era of modern reperfusion therapies.

Ceasing beta-blocker therapy in patients without a clear benefit could streamline treatment, reduce side effects, and lessen the overall burden on patients and health care systems. In addition, it could mitigate the negative effects of polypharmacy and enhance medication adherence. However, before discontinuing beta-blocker therapy, it is critical to undertake a thorough risk-benefit clinical evaluation, similar to the protocols applied to patients with recovered heart failure with reduced LVEF.

## Funding support and author disclosures

The authors have reported that they have no relationships relevant to the contents of this paper to disclose.
